# Topical corticosteroid counselling among Malaysian community pharmacists: a qualitative interview study

**DOI:** 10.1186/s12875-023-02071-z

**Published:** 2023-05-25

**Authors:** Abigail Dayang Nathan, Pathiyil Ravi Shankar, Chandrashekhar T. Sreeramareddy

**Affiliations:** 1 Public Health student, School of Postgraduate Studies, International Medical University, Kuala Lumpur, Malaysia; 2 IMU Centre for Education, International Medical University, Bukit Jalil, 57000 Kuala Lumpur, Malaysia; 3 Division of Community Medicine, International Medical University, Kuala Lumpur, Malaysia

**Keywords:** Counselling, Community pharmacists, Community pharmacies, Malaysia, Topical corticosteroids

## Abstract

**Background:**

Topical corticosteroids (TCS) are commonly available in community pharmacies and are used in skin conditions like atopic dermatitis and psoriasis among others. Problems such as overuse, use of potent steroids and steroid phobia have been identified in the use of TCS in the literature. The aim of the study was to obtain community pharmacists (CPs) views regarding factors influencing their counselling of patients about TCS; challenges associated, important problems, the counselling process, shared care with other healthcare professionals, and explore further the findings from the questionnaire-based study.

**Methods:**

Seven licensed practicing community pharmacists (from the Klang Valley, Malaysia) were interviewed between 23^rd^ September to 14^th^ November 2021. These were CPs participating in the questionnaire study who agreed to be interviewed. NVIVO 11 software was used for data analysis. Codes and themes were generated and agreed on by the researchers.

**Results:**

The major themes identified related to the process mentioned of providing information to patients, the issues addressed by CPs during the counselling (including steroid phobia, overuse of TCS, patients asking for a specific preparation by name), less counselling support material, language barriers, lesser knowledge about certain conditions, information sources used by CPs (material provided by Ministry of Health and Malaysian Pharmacists Association, MIMS) and suggestions to strengthen the quality of counselling (specialization in skin diseases, webinars, shared care models). For patients requesting a particular preparation by name, the pharmacist will decide whether the preparation requested is suitable or suggest an alternative. Steroid phobia was seen more commonly among parents of young children and young patients. MIMS was available as a smartphone application making it easier to use. Advanced training for CPs in the management of skin conditions like that provided for diabetes mellitus can be considered.

**Conclusions:**

Counselling was conducted while dispensing TCS in the open area of the pharmacy. Challenges to counselling were lack of time, limited counselling materials, and language barriers. Steroid phobia requires attention. Initiatives to strengthen counselling were mentioned by respondents and appear feasible. Further research covering the entire country is required.

**Supplementary Information:**

The online version contains supplementary material available at 10.1186/s12875-023-02071-z.

## Introduction

Community pharmacists (CPs) play an important role in the delivery of primary health care through services such as supplying medications, medication review, and medication and lifestyle counselling. Malaysia comprises 13 states (Johor, Negeri Sembilan, Melaka, Perak, Penang, Kedah, Perlis, Kelantan, Selangor, Pahang, Sabah, Sarawak, and Terengganu) and 3 federal territories, (Kuala Lumpur, Labuan and Putrajaya) with the state of Selangor and Kuala Lumpur being densely populated and the nation’s main financial and economic hubs Pharmaceutical care services provided by CPs in Malaysia were considered inadequate and suboptimal collaboration with other providers, less proactive management of patients’ medications, and problems with documentation were noted [1].

Topical corticosteroids (TCS) are a common medication used in healthcare facilities, including community pharmacies [2]. To minimise adverse drug reactions associated with TCS, their use should not exceed 14 days without a physician’s recommendation [3]. Skin conditions account for 20% of primary care visits in Malaysia and TCS are used to treat several skin conditions, including atopic dermatitis (eczema) and psoriasis [4]. Factors such as the formulation and potency of the preparation, the severity of clinical presentation, patient’s age, affected body part, advantages, and adverse effects, influence the choice of TCS [5].

Studies have shown that misuse of TCS is a major problem in several developing countries. A study in Pakistan had shown that pharmacists could assist to a large extent in reducing TCS misuse [6]. Patients were not counselled adequately regarding the proper use of TCS [7]. Comprehensive counselling should help patients use TCS properly and reduce the incidence of adverse drug events (ADE). After counselling patients should be aware of the potency and strength of the preparation, know how to apply it, the side effects that can happen, and the maximum duration of use. They should know when they must follow up with the pharmacist and the parameters to be monitored to reduce ADE and improve the efficacy of therapy.

A recent systematic review concluded that TCS phobia is a widespread and cross-cultural phenomenon and studying it may provide opportunities for interventions to improve treatment adherence [8]. TCS may also be overused and abused. Studies from India indicate widespread overuse, abuse, and misuse of TCS [9]. Super potent products are marketed and strongly promoted, especially to non-dermatologists. Many TCS are available OTC (in India) and are being repeatedly used by laypersons. In India, TCS are applied for a fair complexion, for melasma, to reduce hyperpigmentation associated with acne, and for dark circles [10]. Over 80% of patients had obtained preparations OTC, 8% were influenced by attractive advertisements, 8% used it on the recommendation of friends and family and only 4% had consulted with a dermatologist. In Malaysia, TCS are divided into 4 classes- Class I (very potent)—Class IV (mild), and all are under Regulatory Classification C, meaning they can be dispensed by a pharmacist without a prescription [4]. TCS can cause different types of adverse effects ranging from local adverse effects on the skin, and systemic adverse effects that may be more likely to develop when high potency TCS are used on areas with thin skin like the face or on raw and inflamed surfaces [11]. These can range from hypothalamic–pituitary–adrenal axis suppression, hyperglycaemia and diabetes mellitus, and mineralocorticoid adverse effects.

We did not come across previous studies exploring CPs perspectives on counselling patients about TCS in Malaysia. Counselling about TCS use is crucial due to a variety of issues ranging from steroid phobia to incorrect use of TCS. A Canadian study identified pharmacists as trusted healthcare providers and medication experts who were ideally placed to counsel patients about TCS and reduce their misconceptions [12]. The objective was to investigate CPs views regarding factors influencing the provision of good quality counselling to patients on TCS were investigated. Their views on issues to be addressed during counselling, challenges in providing good quality counselling, and suggestions to strengthen counselling were noted.

## Methods

Study design: Qualitative in-depth interviews were conducted.

Study setting: The study was done in the Malaysian state of Selangor and the two federal territories of Kuala Lumpur and Putrajaya.

Participants: Community pharmacists working in pharmacies located in these three areas as per the eligibility criteria.

Recruitment: CPs willing to participate.

- CPs holding type A licence certification. Type A license is issued to a pharmacist to import, store, and generally deal with all poisons both wholesale and retail or by wholesale or by retail [13].
- Currently working in community pharmacies in Selangor, Kuala Lumpur, or Putrajaya.

CPs were recruited through email, telephone, and social media for a questionnaire-based study (conducted along with the qualitative interviews) and were also asked if they would like to participate in a semi-structured interview with the objectives mentioned before. Despite follow-up and reminders, only twelve CPs expressed interest. The researchers could only contact CPs and request their participation through online methods due to the COVID-19 pandemic and subsequent movement restrictions.

### Data analysis

This study was conducted along with a study on the knowledge and practices of CPs about providing counselling regarding TCS using a questionnaire that has been published [14].

In-depth interviews were carried out to investigate the opinions, challenges experienced, and the role that CPs play in enhancing counselling. The interviews were conducted by the first author from 23^rd^ September to 14^th^ November 2021 by telephone in the English language. The first author was a part-time Master of Public Health student, and a Community Pharmacist (Bachelor of Pharmacy) practicing in the state of Selangor. She underwent training in qualitative research methodology and interview techniques. She established contact with the interviewed CPs and interacted with them prior to the actual interview. She explained to the interviewees that she was a CP and an MPH student doing the research as part of her thesis. The interviewer was involved in counselling patients about TCS and was aware of the challenges faced in counselling. While conducting the interviews, the interviewer was very aware to not pose leading questions and to emphasize that the interview was not a test of the respondents’ knowledge or skills but an opportunity to walk through a typical counselling process and obtain their views on how CPs can improve on counselling, especially with regards to TCS. Field notes were taken by the interviewer, and the interview lasted approximately 30 min. The transcripts were shared with the participants for member checking. The transcripts were read through by the first two authors and the data was reviewed at the conclusion of each interview.

The study is reported in accordance with the Consolidated Criteria for Reporting Qualitative Research (COREQ) checklist (Supplementary file). The researchers employed qualitative description in this study. The perspective of CPs regarding the use of TCS has not been previously studied in Malaysia and qualitative description provides a lesser level of inferences so that it remains closer to the original data [15]. The researchers adopted a pragmatist research paradigm and understood that patient counselling is complex and constantly evolving and should be interpreted against the changing backdrop of pharmacy practice. Information about counselling provided by a CP for other topical medications was available from previous studies. Hence the researchers used a mixture of directed and conventional content analysis to identify the main themes and areas to be explored [16]. A mixture of inductive and deductive approaches was used. Within the main categories, a conventional content analysis process was used to explore categories that emerged from the data.

The final sample size was seven participants, as the researcher saw no new themes emerging after the sixth person. We are aware that this method may require maximum variation sampling to obtain a broad insight into the studied phenomenon, but we were limited by the homogeneity of the CPs, the low response to the interview request, the COVID-19 pandemic, and consequent movement restrictions, and the budget available. Before conducting the interviews, a semi-structured interview guide was developed through a literature review [17, 18].

Following this, a pilot interview was carried out with a participant (a CP with similar characteristics as the study population) selected through purposive sampling. The interviewee provided no negative input on the interview questions. This provided us with confidence that the interview guide can be used among the participants. Interview questions followed the sequence in the interview guide (shown in Additional file 2). The telephone interviews were conducted online and only the interviewer and the interviewee were present. Notes were also taken during the session. The interviews were audio-recorded and transcribed using the Microsoft Office 365 dictation-transcription tool and then edited manually. No repeat interviews were conducted. Transcripts were anonymised prior to further analysis. Interviewees were provided RM20 (approx. 5 USD) e-vouchers. The transcript of the interview was sent to each participant for review, corrections if any and confirmation.

NVIVO 11 software (version 11, 2020) was used to help with coding and data management. The transcripts were read repeatedly to achieve a general understanding and familiarise the researchers with the interview data. The IMU Joint Committee on Research and Ethics (IMUJC) approved this research (Project ID number: MSPH I/2021(04) dated 14 February 2021).

## Results

Seven community pharmacists were interviewed. Three were male, six were in the age group of 21–30 years, five were of Chinese ethnicity, and four were practicing in Selangor. Five respondents had been practicing for less than five years. The major themes that emerged are shown below. The themes were the process of providing information to the patients, major issues addressed by CPs during counselling, challenges faced during the process, sources of information used by the CPs, and suggestions to further improve the counselling quality. Counselling was provided while dispensing, and counselling in a dedicated private area was not provided. Steroid phobia, request for a specific preparation by name, and patient continuing to use the preparation for an extended duration were challenges mentioned. There was agreement among the seven CPs interviewed regarding the major themes.

### Process of providing information to patients

In this situation like in most other developing nations, information and advice is provided by the CP during the process of dispensing. Patients visit the pharmacy and purchase medicines either with or without a prescription. The pharmacist provides information about the medicines including how to take/method of application, adverse effects, and precautions, and when to report back to the healthcare professional. Due to the number of patients visiting the pharmacy and the difficulty in billing for their counselling services patients were not counselled in a private counselling room. This was mentioned by all CPs. Most patients visited the pharmacy asking for a TCS preparation OTC. Hence, there may be differences in the use of the terms ‘counselling’ and ‘dispensing’ in the current study as compared to international usage. CPs mentioned that they do not rush the patient and they address all the doubts and concerns the patient may be having.

CPs examine the skin condition carefully and then decide if drug treatment in the form of TCS is required or only a moisturizer. Patients may walk into the pharmacy with the request for a specific brand name skin preparation. In this case, the CP examines the skin carefully and then decides whether the requested preparation is suitable, or an alternative may be provided. They educate patients about the strength and potency of TCS. The method of application is demonstrated, and patients are warned not to apply the preparation for more than two weeks.

“I will explain to them the pros and cons of using the steroid medication and of course I also always without fail always provide a bit of education with regards to the strength of the steroid and whether it's suitable for their condition or not.” (CP4).

A similar process was followed by all CPs.

### Major issues addressed by CPs during counselling

CPs face three main issues while counselling patients regarding the use of TCS. The first is steroid phobia, the second is the patient continuing to use the preparation for an extended duration and the third is demand for a specific preparation usually based on advice obtained from friends and relatives. Steroid phobia is especially common among parents of young children and younger persons who obtain information from the internet. CPs inform patients about the difference between oral and topical steroids. Information on the potency of the steroid and on the strength of the preparation is provided. The method of application is mentioned. Differences in skin thickness at different locations in the body necessitating different preparations and the correct method of application are stressed.

“One is they confused the topical steroid with the oral steroid. That one is quite easy to debunk. But the other thing is, they would already know that the steroid will cause skin damage, so we need to explain about the side effect and time, don't use it for quite long, things like that. But another type of patient is a bit harder because they already (have) a phobia of the cream. Again, we explain only. If they accept, then accept. If they don't accept, there’s nonsteroidal cream in the market.” (CP3).

“Steroid phobia I can say mostly I've encountered is like a young, younger adult because probably due to like Internet they have (done) Google searches like steroid is not good. For young parents when they show us their kids’ condition, they really need steroid to apply and they say oh, is it possible not to use that? Usually, I'll say for some skin conditions will still need steroids to really recover, just that don't use it long term. It will be OK.” (CP1).

In their interviews, all CPs mentioned steroid phobia and its effect on choosing an appropriate treatment.

Elderly patients may sometimes continue to apply a skin preparation prescribed by their doctor as they had obtained relief after using it and they believe that if it was prescribed by their doctor then it should be good. The CP advises them to consult their doctor again and educates them about the side effects that can occur due to continued use of TCS. Three respondents mentioned this during the interviews.

For patients who walk into a pharmacy requesting a specific steroid, the CP tries to educate them about their skin condition, the potency, and strengths of different preparations, and the one that may be most suitable for their condition.

Two respondents specifically mentioned that they only provide advice and try to convince the patient but if the patient decides not to follow their advice then the requested preparation may be dispensed.

### Challenges faced during counselling

Though CPs counsel about dermatological conditions and about the different TCS available there is no formal separation of counselling from the process of dispensing. There is a lack of suitable visual material though this is now partly addressed by images obtained from the internet and by the material provided by the Malaysian Ministry of Health and the Malaysian Pharmacists Society. There may be language barriers and though this is partly addressed using online translation some patients prefer to consult with a pharmacist of their own race. These issues were highlighted by three respondents.

“The language barrier here I meant for certain Chinese-speaking customers only. Where they either only understand Chinese, or they opt to only listen to Chinese-speaking pharmacists. Personally, for me due to the place I’m working, I receive a lot of expatriates that speak their native (language) again like from China and Bangladesh to them to speak I need the help of Google Translate. Some are eager to cooperate, but some just get offended and walk off or demand certain drugs. So, we had to deliver without proper diagnosis and counselling.” (CP5).

CPs also mentioned that they may lack knowledge about some skin conditions and regarding conditions that do not respond quickly to treatment. This issue was mentioned by four respondents during the interviews.

“I would be more comfortable if their skin conditions are very mild, and not that very extensive, like to the point that it's like psoriasis, I would feel a bit reluctant, because of course I am not trained in dermatology” (CP4).

### Dispensing medicines prescribed by doctors

In Malaysia usually, doctors also dispense the medicines prescribed by them and there is a lack of dispensing separation. If the preparation is not available then the patient may visit a pharmacy. In this case, the CP checks the information provided by the physician and most respondents mentioned that the doctors counsel their patients very well.

There usually is not much communication between doctors and CPs, though two respondents mentioned that they have been able to establish contact with local doctors and check with them if they have any queries about their prescriptions.

### Sources of information used by CPs

CPs used the material provided by the Malaysian Ministry of Health and the Malaysian Pharmacists Society. They also have online groups consisting of their batchmates and seniors at pharmacy college and this is used to share information. A pharmacist who had worked previously at the Ministry of Health still maintains contact with his colleagues. They also attend continuing education programs and webinars. The Monthly Index of Medical Specialties (MIMS) was mentioned as a commonly consulted source, and this is available as a smartphone app. Five of the CPs mentioned using these sources.

### Suggestions to further improve the quality of counselling

The importance of shared practice with the physician prescribing medicine and the CP dispensing the same was mentioned by all pharmacists. Lack of time was mentioned as a major reason for not establishing contact with other healthcare professionals.

“I feel like in Community pharmacy we have like a bit terpulau (isolated). Meaning, if they want it, they come to us. We don't have a basic communication between all of us.” (CP5).

In Malaysia, CPs have been provided advanced training in the management of diabetes mellitus, and CPs who see many patients with skin conditions want a similar system to be established for skin diseases. They want to collaborate with a dermatologist who could educate them about the diagnosis of common skin conditions requiring the use of TCS, and conditions that may need a referral to a physician. A process of certification of CPs in the management of skin diseases can be initiated.

“If MPS [Malaysian Pharmacists Society] itself or maybe just a few pharmacists, it can produce a short PDF form for proper use, I think that would be very useful. Although we already have the Topical corticosteroids preparation counselling by the KKM [Malaysian Ministry of Health], I don't think everyone has it, so maybe we can make it available once again, so at least not all of us would wrongly diagnose or treat it” (CP5).

Two CPs mentioned that information can be posted in private social media groups regarding TCS and other medicines and about the diagnosis of different skin conditions. Table 1 shows additional comments by the CP respondents under each theme.

**Table 1:** Additional comments by the CPs under each theme

Theme	Supporting statements
Process of providing information to patients	• So, what kind of range of skin condition people come for actually ranges from eczema, which I feel there is a need for corticosteroids. Some actually just come just because of insect bite, and they demand for corticosteroids and also some people who just have like simple rashes, they'll just say like, oh, I used to use these. So, I want it at the kind of skin conditions they will just come and ask for it. (CP5)
Major issues addressed by CPs during counselling	• I will explain to them the pros and cons of using the steroid medication and of course I'm, I also always think I always without fail I always provide a bit of education with regards to the strength of the steroid and whether it's suitable for their condition or not. (CP4) • So, let's say in terms of noncompliance. Maybe they don't, the patients don't really tell me why they don't use it, but I would try to explain to them that it's necessary to for the symptoms to subside by using these medicines. So sometimes, maybe the patient might feel, because it's a steroid and because it has a bad reputation among their circle, so they refused to use it. Or on the other end when they overuse it, they usually don't realize that they shouldn't use it for extended periods of time. (CP2)
Challenges faced during counselling	• Normally it's better if we can bring them to a consultation room and then ask all the necessary questions for the condition that to get more in depth understanding. But not feasible. It would be good to do that, and I spend some time asking all the questions related to the skin and then then we can even recommend a more appropriate cream like based on the potency and things like that and have a better consultation but. Yeah, but not feasible. (CP3)
Sources of information used by CPs	• Product leaflet also got but this one. When you study in your university, you should know already. Basic basic. Or you can use KKM (Malaysian Ministry of Health) guideline, the counselling guideline. (CP6)
Suggestions to further improve the quality of counselling	• For this topic, I would say, I really hope MPS, I really hope like we shall deliver each other to each other a better knowledge. So perhaps it can start from myself. Maybe I can start sharing in my in my media. Social media about all this pharmacy related stuff that. So, let's just grow together. (CP5)

The supplementary file shows the analysis of the study according to the COnsolidated criteria for REporting Qualitative research (COREQ) checklist.

## Discussion

The major issues noted about TCS are steroid phobia, overuse of the preparations, and patients asking for (strong) preparations by name. There are also challenges in terms of diagnosing the skin condition, counselling aids, and language barriers. Communicating with other HCPs was a challenge. Different information sources were used and additional training for CPs in terms of managing skin conditions was recommended.

Steroid phobia was a major problem mentioned. Steroid phobia was a critical concern raised, especially among parents of young children, patients who had used steroids before. In Malaysia, as in other countries, community pharmacies are concentrated in urban areas. In the present study, CPs mentioned that while they would try to educate the customer if the requested TCS is inappropriate for the condition presented, they would still comply with their customer’s requests should they insist. Pharmacist-provided counselling is a value-added service and in a study conducted in the state of Sabah, Malaysia over 55% of respondents mentioned that they are willing to pay for counselling provided by the pharmacist and the median price they were willing to pay was RM 5 [19]. CPs do not charge for TCS counselling at present, in the country.

Dispensing, and counselling patients about TCS is challenging. CPs did not consistently provide complete counselling information and they mentioned that in certain cases this was done not to increase the patients’ steroid phobia further. There may also be a lack of time during consultation to explore the subject matter. This lack of time may be due to the maldistribution of community pharmacists in Malaysia and their concentration in large cities as mentioned previously. Malaysia has approximately 2,889 community pharmacies, and 3,892 registered community pharmacists across the country [20]. This shortage is more evident in rural areas, where there is a lack of pharmacies [21]. The requirements for a counselling area in Malaysia have been defined by the Community Pharmacy Benchmarking Guideline [22]. The medicine dispensing area must be a minimum of 18 square meters (193 square feet) in size and consists of the dispensing counter and counselling area. The counselling area does not need to be in a private room if the area is comfortable and suitable for effective communication with the patient. Most respondents counsel patients during the process of dispensing and do not use a separate counselling area.

The issue of the language barrier was also brought up. CPs described the challenges they face in counselling patients who do not speak English or Malay. This concern was also reported in a study of community pharmacists in East Malaysia [23]. Participants used mobile applications such as Google Translate to aid in communication. A systematic review mentions that language barriers hamper communication between healthcare professionals and patients, decrease satisfaction and impair healthcare delivery quality and patient safety [24].

CPs mentioned strategies to ensure patients’ safe and effective use of TCS including education and policy efforts, cooperation among healthcare professionals, and initiatives by individual CPs. They proposed training modules, the opportunity to specialise in dermatological conditions, and increased access to guidelines. Improving pharmacists’ dermatological competency will benefit patients by improving treatment outcomes and addressing the underuse, misuse, or abuse of TCS. CPs have been trained to provide care for various conditions in Malaysia. A train-the-trainer program for CPs on cardiovascular health was organized and increased confidence and ability to conduct workshops for other CPs were noted among the participants [25]. A targeted training program using a diabetes tool was offered to CPs and the training improved their diabetes management knowledge [26]. Hence offering training programs and further certification to CPs interested in managing dermatological conditions should be feasible. A detailed counselling guide for pharmacists on topical preparations has been prepared by the Pharmaceutical Services Programme of the Ministry of Health, Malaysia, and is freely available online [27]. Pages 23 to 26 of the guide deal with TCS. Information about potency of steroids, counselling points, adverse effects and fingertip unit of administration is provided.

Additionally, participating CPs proposed initiatives to promote the safe and effective use of TCS. Renewal of the Annual Retention Certificate for the purpose of practicing as a pharmacist necessitates obtaining continuing professional development (CPD) points via seminars, workshops, or skills courses [28]. Social media has been used to educate pharmacists. A systematic review found that pharmacists used Facebook as a means of professional networking and for collaborating with colleagues [29].

This study has several limitations. First, this study was conducted only among a limited number of CPs in the state of Selangor, and the Federal territories of Kuala Lumpur, and Putrajaya. Second, the clinical outcomes of patients, which would inform about the impact of pharmacists’ counselling in the treatment of dermatological conditions were not evaluated. The participants’ perceptions and behaviours were self-reported, and this may lead to response bias. The respondents were from pharmacies located in urban areas and so may not be representative of rural pharmacies. These limitations can affect the generalizability of the findings.

## Conclusions

This study identifies important issues regarding counselling provided by CPs about TCS. Steroid phobia, request for a specific preparation and overuse of TCS were mentioned as problems. Lesser number and types of counselling aids, lower confidence in treating certain skin conditions, and the challenge of updating knowledge were mentioned. Training programs using the guidelines for use of topical preparations [27] can be conducted. Shared management of patients, provision of management guidelines of common skin conditions, advanced training in managing skin conditions, and use of social media groups to provide the latest information were mentioned. Further studies in other parts of the country are required.

## Supplementary Information

**Additional file 1.** COREQ (COnsolidated criteria for REporting Qualitative research) Checklist.

**Additional file 2.** Semi-structured interview guide for the interviews with the pharmacists.

## Data Availability

The datasets generated during and analysed during the current study are not shared publicly to preserve the anonymity of the respondents but are available from the corresponding author on reasonable request.
