# Supplementary material for: Topical corticosteroid counselling among Malaysian community pharmacists: a qualitative interview study
Source: BMC Prim Care. 2023 May 25;24:119. doi: 10.1186/s12875-023-02071-z (PMC10211295; doi:10.1186/s12875-023-02071-z)
Supplement: Supplementary file 2 — Additional file 2. Semi-structured interview guide for the interviews with the pharmacists. [file 12875_2023_2071_MOESM2_ESM.docx]

**Additional file 2: Semi-structured interview guide for the interviews with the pharmacists**

**Topics**

| **Main topic** | **Subtopic** |
| --- | --- |
| Drug-related problems | Treatment problems  Handling of problems  Opinions about the use of TCS (perception, steroid phobia) |
| Patient counseling and information needs | (Type of) information provided from the pharmacy  Suggestions in improvement in information provision |
| Collaboration with other health care provides | Collaboration with other health care providers (agreements, information exchange)  Consistent communication between doctor and pharmacist |
| Educational needs | Knowledge and skills of the pharmacy team  Education needs of the pharmacy team  Knowledge and skills of pharmacy team according to the pharmacist |

**Questions posed during the interview**

| **Main topic** | **Subtopic** |
| --- | --- |
| 1. Drug-related problems | - What are the problems you face when treating a patient presenting with skin issues? - How do you handle those problems? - What are your opinions on the use of topical corticosteroids for the treatment of skin problems? (Perception, steroid phobia, etc.) |
| 1. Patient counseling and information needs | - What are the types of information provided at the pharmacy regarding skin problems? - Do you have any suggestions to improve the provision of information to patients? |
| 1. Collaboration with other health care provides | - How do you counsel a patient who has been prescribed a topical corticosteroid? - What are the information sources available in your pharmacy? Which ones do you commonly use? - How do you know an information source is objective and reliable? - What do you think about collaboration with other health care providers? (e.g. Information exchange) |
| 1. Educational needs | - How do you think we could improve the knowledge and skills of a pharmacist and their team regarding skin conditions and their management? |
